# Case report: Extreme respiratory sinus arrhythmia in a non-athlete female student - a peculiar finding at the Physiology practicum

**DOI:** 10.3389/fnins.2024.1507269

**Published:** 2024-11-29

**Authors:** John M. Karemaker

**Affiliations:** Department of Medical Biology, Section Systems Physiology, Amsterdam University Medical Centers, Amsterdam, Netherlands

**Keywords:** vagus nerve, cardiorespiratory interaction, evolution, respiratory gating, baroreflex, heart rate, sinus node, paced breathing

## Abstract

During an ECG-training course, a case of extreme respiratory sinus arrhythmia (RSA) was found in a 19-year-old slender, female student who was not active in sports. The heart rate (HR) fluctuated from above 100 to below 60 beats per minute (bpm), often from one beat to the next. The pattern was repetitive and appeared to be linked to respiration, representing an extreme form of RSA. The initial recording of the HR and blood pressure (BP) by finger blood pressure showed concomitant drops in diastolic BP of up to 25 mmHg. The student agreed to participate in a short follow-up study, during which HR, BP, and respiration (measured by temperature and pCO_2_ of the airflow at the nose) were recorded in the supine and upright tilted positions. Measurements were taken during 5 min of rest, during paced breathing (1 min each at 6, 10, and 15 breaths per min), and during end-expiratory breath-hold. This study presents a beat-by-beat analysis of the observed interrelations between respiration, HR, and BP. Her respiratory rate with maximal RSA was found to be only slightly lower than the spontaneous rate, at 10 versus 12 breaths per min. From the combined observations, it was concluded that, in this case, the baroreflex relationship between spontaneous BP and HR changes was overridden by near on/off gating of (possibly massive) cardiac vagal outflow. This is due to a central, respiration-coupled gating mechanism, with the vagus nerve being “on” during expiration and “off” during inspiration. Such a system will destabilize blood pressure. It shows an evolutionary remnant of optimizing lung perfusion during air inflation, regardless of the consequences for systemic blood pressure.

## Introduction

**Figure 1:**
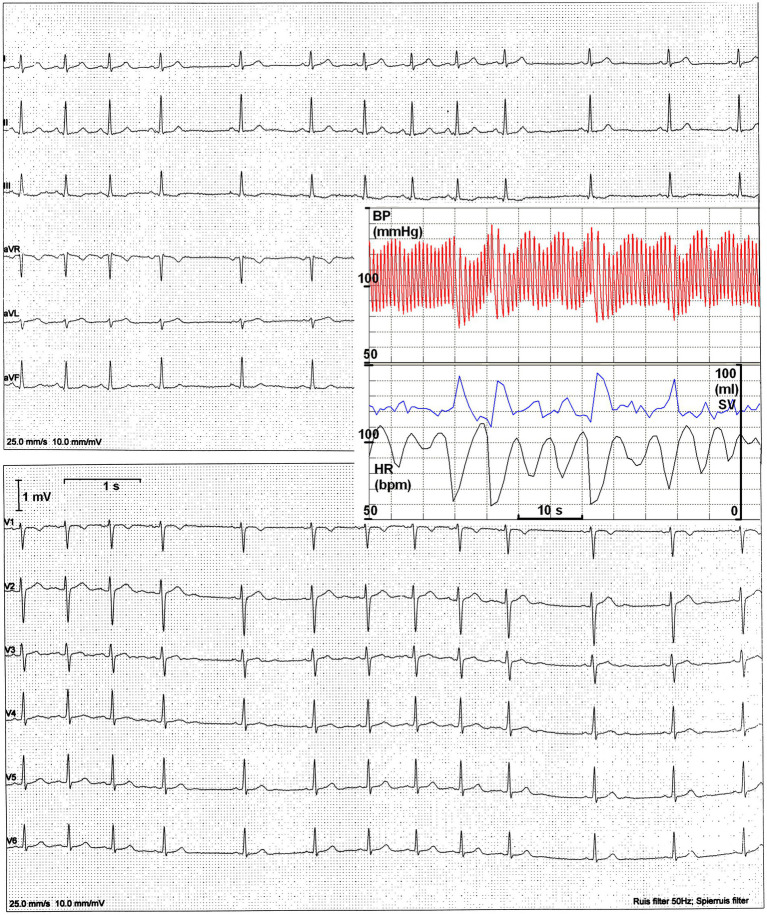
The original 12-lead ECG. Heart periods range from 580 to 1,120 ms. Insert: finger BP recording with simultaneous evaluation of HR and estimated stroke volume (SV).

The recording of a 12-lead ECG by second-year medical students for each student in their group is part of our Physiology course. Fourth- and fifth-year students assist in the procedure after being trained to convey the set learning points. Staff members are available for help and support. One particular ECG was brought to my attention for diagnosis: the assistants discovered an unusual arrhythmia and were unsure how to interpret it. The recording showed heart rate (HR) changes, fluctuating within one or two beats from above 100 to below 60 bpm (Figure 1). Inspection revealed that these variations were repetitive and likely due to respiration. This explanation was provided to reassure both the student group and the assistants.

To complement the ECG recording, she was later connected to a finger blood pressure (BP) device while seated in a quiet environment for concurrent BP oscillations (Figure 1, insert). Clearly, a longer heart period was followed by a larger stroke volume; however, this did not compensate for the increased diastolic runoff, resulting in a decline in pressure.

In the interest of science, I proposed a short follow-up study to investigate the relationship between respiration rate, BP, and HR while she was supine and standing (in a relaxed upright tilted position). This report provides an account of that study. The results of the follow-up study place respiratory sinus arrhythmia (RSA) within a broader context of evolution and economy of circulatory function in relation to respiration. This case has been presented at several meetings (without any official publications) to provoke discussion and in the hope of finding similar cases; however, this effort has been in vain.

## Methods

### Medical ethics

Given the teacher–student relation, special permission for this study was obtained from the Faculty’s Committee for the Examinations. The research protocol was approved by the Medical Ethics Committee of the Medical Faculty of the University of Amsterdam. Written consent was obtained after providing full oral and written information, along with sufficient time for the student to consider her participation, in accordance with the Declaration of Helsinki.

### Test participant’s data

The student was a 19-year-old woman in good health. She was a non-smoker and not taking any medication (height:163 cm; weight:52 kg; and BMI: 19.6). For many years, she was known to have cold hands and feet; however, this condition did not affect her normal daily activities. She was not active in any sports apart from cycling, which she used as her usual mode of transport for short distances. Earlier, during high school physical education classes, she was noted to have “high heart rates” (unspecified). For the also observed ‘irregular heart rate’ she had been referred to her GP who had assured her that everything was normal.

### Test procedures

She arrived at the lab in a post-prandial state, more than 2 h after a light breakfast, and no caffeinated drinks had been consumed. While she was supine on the tilt table, ECG electrodes were applied for a single-lead thorax ECG and a finger cuff for continuous blood pressure recording via a Finometer® (FMS, Amsterdam, Netherlands). For respiration recording, we fitted a nose probe connected to a sampling capnograph (HP, 1436A) and a temperature probe (Nihon Kohden) to accurately monitor inhalation and exhalation. The temperature signal was high-pass filtered to eliminate drift. All data were digitized online and stored for later offline analysis.

Initially, tryouts of the tilt, breath-hold, and paced breathing maneuvers were performed. The end-expiratory breath-hold was performed with an open glottis to prevent a Valsalva maneuver. Paced breathing was performed by following a tone that ascended for inspiration and descended for expiration (40% and 60% of the cycle respectively to inspiration and expiration). Lights indicated hypoventilation or hyperventilation. After sufficient training, data collection began with a 5 min supine resting period, followed by a 20-s breath-hold. This was followed by paced breathing in 1-min intervals at 10, 6, and 15 breaths/min respectively, interspersed with 1 min of free breathing. Then, the table was tilted to a 70° upright position. After 5 min of relaxed upright standing, the breathing protocol was repeated. After tilting back to supine, another 2 min of relaxed recording followed, concluding the experimental protocol.

### Data analysis

Beat-to-beat values for heart period, systolic, diastolic, and mean blood pressure were measured offline. RSA was read from the heart period excursions for each breath during the specified periods. The heart periods were mostly derived by the software from the blood pressure signal; therefore, interbeat intervals (IBIs) are reported, unless the R-waves were used for more accurate timing of events in the cardiac cycle.

Cardiorespiratory coupling analysis was performed manually due to the subtle variations in the temperature recordings at the nose. Using Acrobat Pro™, the distance between the R-wave and the first observable change in the temperature was measured from the screen, whether downward (inspiration) or upward (expiration). The concurrently measured capnogram served as confirmation, but it was not used for timing. Due to the delay time of the sampling capnograph (about 660 ms) and the flushing of the airway’s dead volume during expiration, exact timing from that signal is less reliable.

## Results

### Resting recordings: supine and upright tilted

**Figure 2:**
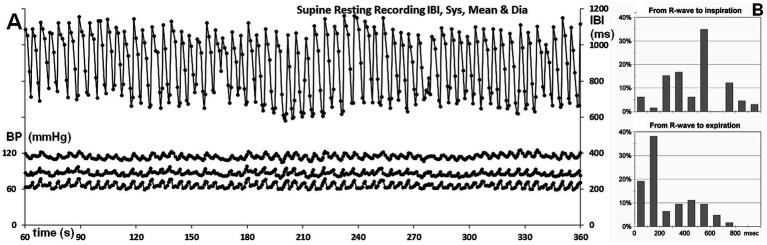
**(A)** Overview of the supine resting recordings; IBIs are shown on the right-hand scale, while systolic, mean, and diastolic finger pressures per beat are displayed on the left-hand scale. Timescale: time during the experiment. **(B)** Percentage of occurrences for the time from the R-wave to the start of inhalation or exhalation, shown at the top and bottom, respectively.

**Figure 3:**
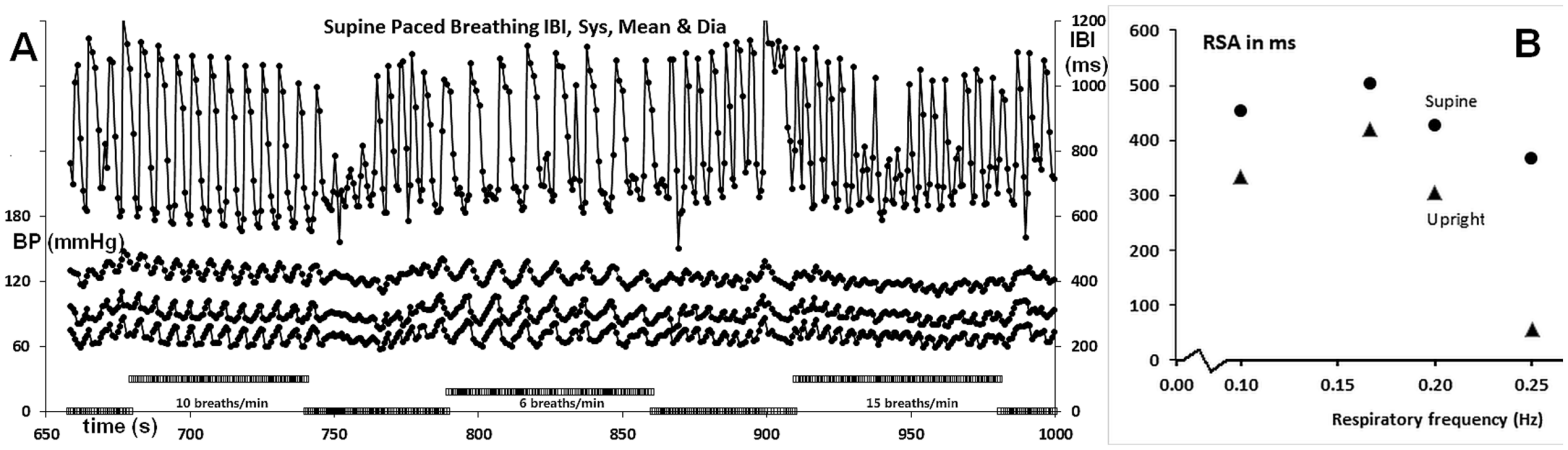
**(A)** IBI and BP recordings during paced breathing, showing 10, 6, and 15 breaths/min from left to right, as indicated by the bars on the abscissa. The axes are the same as in Figure 2. **(B)** RSA as a function of respiration frequency, expressed as ms peak to valley. The values are for paced breathing, except for those at 0.2 Hz (12/min), which are from spontaneous breathing.

The 5-min supine resting period demonstrated remarkable regularity in BP, though RSA showed less consistency (Figure 2A). In the upright tilted position, both BP and IBIs were very variable. For the upright posture, only the first 2 min were taken into account, as the participant appeared to be in an unstable condition afterward. These numbers were used for the curves relating RSA to respiratory frequency (cf. below, Figure 3).

For the supine position, it is important to assess whether there were signs of cardiorespiratory coupling. This is assessed by the ratio between the number of respirations and heart periods [the “whole number ratio,” cf. (Weiss and Salzano, 1970)]. Counting the number of heartbeats between inspirations revealed that 5–6 beats/breath were the prevalent numbers, with similar numbers observed for expirations. Though not exactly a whole number ratio, given the large RSA, one short beat of around 600 ms might flip in and out the respiratory cycle of 5,000 ms (around 12 breaths per min).

This prompted me to track the recordings during spontaneous breathing for more hidden signs of cardiorespiratory coupling, such as the timing of the start of inspiration or expiration in the cardiac cycle (Larsen et al., 2010). The distances from the R-wave to the start of inspiration or expiration are shown in Figure 2B, with the coupling of inspiration displayed at the top and expiration at the bottom, represented as a percentage of occurrences.

If inspirations and expirations had started randomly in the heart period, more or less flat distributions for the starting times would be expected. As a bin width of 100 ms was chosen, this would result in 10% per bin for R-wave to inspiration histogram, with inspirations occurring in intervals of approximately 1,000 ms, and in 17% for R-wave to expiration histogram, with expirations occurring in heart periods of approximately 600 ms duration.

The histogram of inspiration timing within the cardiac cycle shows a sharp peak in the 400–500 ms bin, representing 33.33% (21 out of 63 occurrences). The calculation of the binomial probability of 21 occurrences out of 63 in exactly one bin out of 10 yields a *p*-value of 0.0631, which is not considered significant at the *p* < 0.05 level. However, the literature on cardiorespiratory coupling has previously observed a predilection for the start of inspiration to occur in the middle of the cardiac period (Sobiech et al., 2017). The probability of the maximal number of inspirations occurring in the middle of the heart period, rather than anywhere, is lower than a *p*-value of 0.0631. If we consider the middle three bins, this probability is roughly one-third of that, making the observation significant at the 0.05 level.

Of all expiratory movements, 58% (36/62) started in the first 200 ms of the heart period. This is greater than what could be expected by chance (*p* = 0.0346, with 2 bins to be combined). Expiration triggered a series of long intervals, halting the ongoing interval shortening that occurs during inspiration (cf. Figure 1, insert). Remarkably, when expiration began in the first 200 ms of the interval, the interval immediately following the one in which expiration occurred was already prolonged in one out of three cases. If expiration occurred later, this prolongation was observed only after an additional (short) interval.

In this study, the timings of inspiration and expiration were assigned based on the movement of air at the nose, detected by temperature changes. In the literature, such as in Sobiech et al. (2017), respiratory timings have been derived from various respiration-related signals, such as thoracic movement. Each of these signals has its own time distance from the neural signals that cause respiratory muscle contraction and its mechanical effect.

### RSA as a function of respiratory frequency

Figure 3A displays the full recording of the IBIs and BP during supine-paced breathing, while Figure 3B illustrates the amplitude of RSA as a function of respiratory frequency. The values at 0.2 Hz (5 s/breath or 12 breaths per min) were derived from the resting recordings. The upright values are slightly lower than the supine values, except for the 0.25 Hz frequency (6 ms peak to valley compared to 365 ms). Remarkably, the peak of the curve was found at 0.167 Hz (10 breaths/min) rather than at 0.10 Hz, as reported in most studies, including those by Angelone and Coulter (1964) and Eckberg (1983). However, a higher breathing rate as optimum would align well with the slender build of this test participant (BMI 19.7, height 163 cm). A closer inspection of the recordings during the three paced breathing exercises demonstrates that only the 10 breaths/min respiratory cue was well followed. At the lower 6 breaths/min frequency, abortive inspiratory movements occurred post-expiration, while the highest frequency of 15 breaths/min did not establish a smooth respiratory rhythm. The main difference in amplitude between 6 breaths/min and 10 breaths/min lies not in the longest intervals reached during expiration but rather in the shortest intervals during inspiration.

### Response to the breath-hold maneuver

The supine breath-hold maneuver was performed as instructed: at end-expiration with an open glottis. The upright maneuver appeared less stable, resulting in inconsistent HR responses. During the supine breath-hold, HR stabilized around 60 bpm with 1,000 ms intervals. This duration corresponded to the heart periods that occurred during expiration, confirming the impression that the heart periods ranged between two extremes: 600 ms during inspiration and 1,000 ms during expiration.

### Integration and tentative explanation of the findings

**Figure 4:**
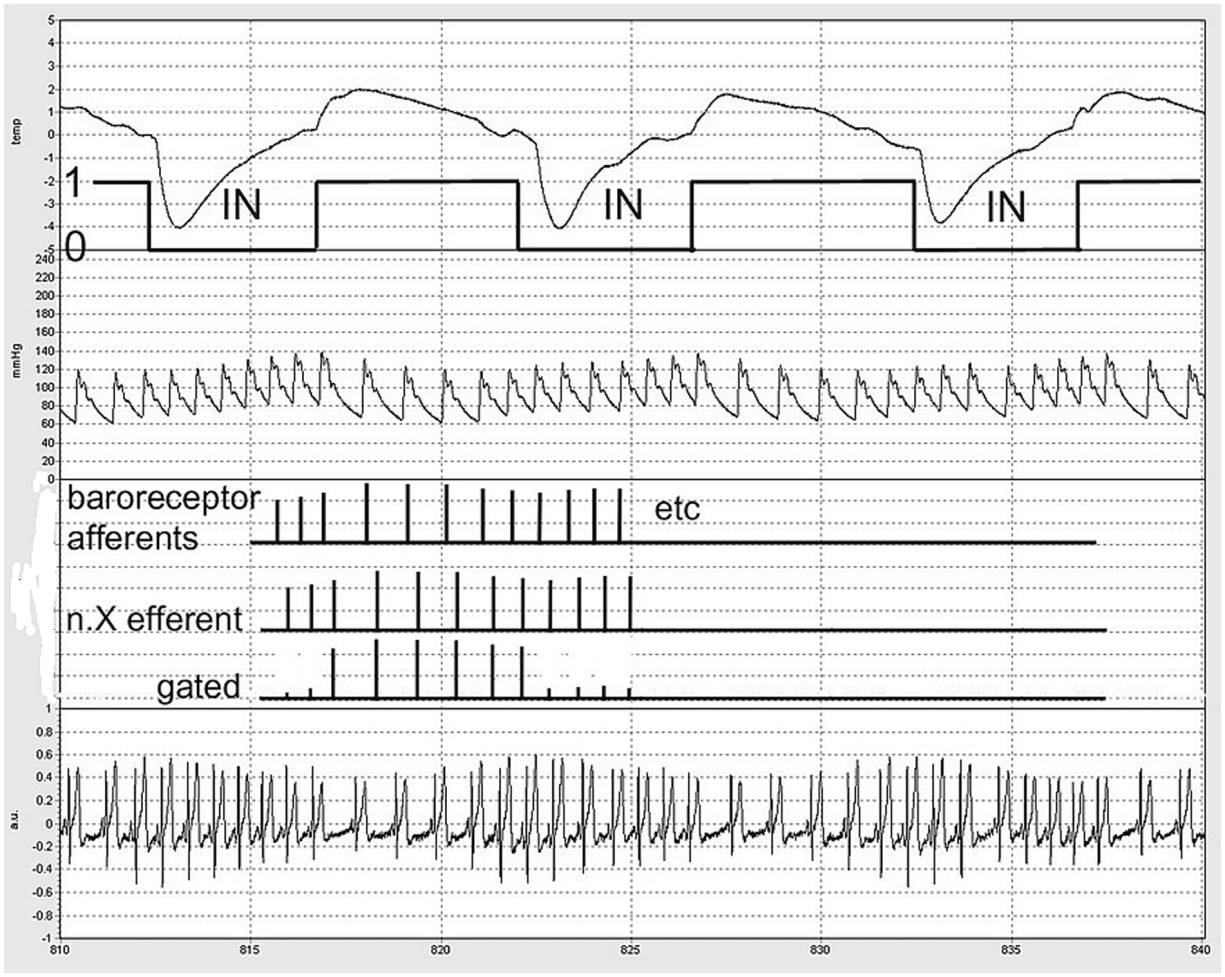
Schematic of ‘central gating’ of vagal outflow by inspiratory movement (labeled ‘IN’). A part of the actual recording was chosen for demonstration. From above down: nose thermistor signal, down: inspiration. Second trace: BP from the finger, third: schematic of baroreceptor afferent firing, n.X: reflex vagal efferent firing without and with gating. Fourth signal: the precordial ECG as measured. Timescale: seconds in the protocol (5 s markings).

The most likely explanation for the observed phenomena is the suppression of outgoing cardiac vagal activity during inspiration, followed by the release of outgoing bursts immediately upon expiration (Figure 4). This aligns with earlier neurophysiological observations supporting a significant central origin of RSA, as noted in the findings of Dergacheva et al. (2010).

Although the supposed “gating” may be central, the signals being gated are of peripheral origin: baroreceptor afferent information and other signals coupled with respiration and the heartbeat that may lead to reflex cardiac vagal outflow were it not for the inhibition caused by inspiratory activity (Dergacheva et al., 2010). In this respect, the term ‘respiratory gating’ is somewhat misleading; it is a balance between inhibitory and excitatory inputs to the cardiac vagal premotor neurons (McAllen and Spyer, 1978).

The combination of these two factors—expiration driving interval prolongation and cardiac contractions driving inspiration—creates a system in which the entrainment of the two rhythms, cardiac and respiratory, may occur when the timings align (Larsen et al., 2010). During the inspiratory period, cardiac intervals are roughly 600 ms in duration, which is too short to allow an immediate vagal effect on the ongoing beat when expiration would ‘open the gate.’ For this effect to occur, intervals must exceed approximately 750–800 ms (Borst and Karemaker, 1983; Pickering and Davies, 1973). However, as mentioned above, if expiration begins very early in the cardiac interval, the next heartbeat could already experience the full vagal effect.

## Discussion

Beat-to-beat heart rate variability is significantly related to respiration (Billman et al., 2015; Buchner, 2011). The term RSA refers to the fact that, despite the “arrhythmia,” each heartbeat still originates from the normal pacemaker of the heart, the sinus node. Under vagal control, HR increases during inspiration and decreases during expiration. The deeper and slower the respiration, the larger the HR excursions (Angelone and Coulter, 1964; Eckberg, 1983). As BP-variations accompany these changes—decreasing during inspiration and increasing during expiration—a baroreflex origin of (part of) this vagal activity has been postulated (Karemaker, 2009). The present case is exceptional in this respect, as the baroreflex appeared to be inhibited during inspiration, while cardiac vagal outflow was overwhelmingly present during expiration.

The concept of central gating aligns well with neurophysiological experiments, although it may not always be as pronounced as observed in this young woman. In a previous study, we applied short stimulation bursts directly to the carotid sinus nerves in humans via their implanted “baropacer.” We found that stimulation during inspiration was, indeed, less effective compared to expiration; however, it was only diminished rather than completely blocked (Borst and Karemaker, 1980). Possibly, this difference in the central processing of cardiovascular and respiratory information was due to the age of our patients, all of whom were middle-aged and using the implanted device for their angina pectoris. It has been shown (Hrushesky et al., 1984) that RSA decreases by more than half from the 20–35 age group to the 65–82 age group, making the inference regarding the effect of age on baroreceptor afferent stimulation plausible. The test participants in the studies surveyed by Eckberg in his review of the evidence for a respiratory gate (Eckberg, 2003) were generally much younger than the baropacer patients.

### Evolutionary origin of RSA

Evolution shows many examples of RSA that are same as or even larger than that recorded in the present case. In some of these instances, the reason for extreme RSA is quite obvious. For instance, overwintering black bears may have their HR rise from a resting 6 to 59 beats/min during a sparse bout of inspiration (Laske et al., 2010). There is recent evidence indicating that this increase in HR is due to the temporary lifting of a strong vagal restraint (Tøien et al., 2024).

Even early in vertebrate evolution, there is a coupling between HR and respiration. RSA has been observed in fish, where water ingestion and gill movements take the place of inspiratory activity (Taylor et al., 1999). They describe how changes in HR adapt the circulation to the moments of optimal gill perfusion when fresh water is pushed along the delicate membranes. This trait is evident throughout evolution, with inspiration favoring increases in HR and lung perfusion, optimizing the effects of the respiratory action. This increase in HR is not due to sympathetic activity but the suppression of cardiac vagal output, as observed in the present case.

### Possible causes for the extreme RSA in this case

The comparison may seem inappropriate, but the RSA presented here closely resembles the normal HR and BP behavior observed in large dogs, as recently reviewed by Grosso et al. (2021), where BP decreases due to a reduction in HR (Figure 1, insert). In general, cardiac vagal outflow varies across the animal kingdom, in part depending on whether the individual in question is a predator or prey.

As the probable mechanism behind RSA—central inhibition—is not peculiar, several questions arise: what causes the vagal effect to be so extreme in this subject? Are there uncommonly large outgoing vagal bursts during expiration? Is the sinoatrial node very sensitive to the vagal influence? Is this part of a hereditary complex involving cardiac vagal activity?

The last question required a follow-up investigation, which was declined. The question regarding the sensitivity of the sinoatrial node to vagal control could not be definitively answered from the present data. However, since no obvious changes in P-wave morphology or PQ-time were detected between inspiration and expiration, this seems unlikely as an underlying mechanism. Additionally, the U-waves observable in the precordial leads V2–V5 (Figure 1) offered no clues. This suggests that the vagus nerve acts as a circumstantial perpetrator, driving resting HR between two extremes: around 100 bpm (with little or no vagal or sympathetic activity) and 55 bpm (at maximum vagal activity). In the supine position, no sympathetic involvement was detectable; however, when sitting (Figure 1, insert) or standing, there was evidence of some sympathetic involvement. This is indicated by the slightly higher maximum HR (shorter heart period) observed.

## Conclusion

A case of extreme RSA was observed in a young, untrained woman. The heart period oscillated between approximately 600 ms during inspiration and 1,100 ms during expiration. The prolonged heart periods during expiration led to an instantaneous BP drop of 25 mmHg. HR and respiration entered a loose coupling, with inspiration being triggered by the heartbeat and expiration activating the previously suppressed vagal cardio deceleration. RSA was primarily centrally mediated, likely due to the inhibition of the baroreflex by inspiratory activity. Maximal RSA excursions were observed at a breathing frequency of 0.167 Hz (10/min), which aligned with her slender build. No cause for the extreme heart rate excursions was identified. No medical follow-up was deemed necessary.

## Data Availability

The raw data supporting the conclusions of this article will be made available by the authors without undue reservation to any reasonable request.
